# (−)‐Phenserine tartrate (PhenT) as a treatment for traumatic brain injury

**DOI:** 10.1111/cns.13274

**Published:** 2019-12-11

**Authors:** Nigel H. Greig, Daniela Lecca, Shih‐Chang Hsueh, Carlos Nogueras‐Ortiz, Dimitrios Kapogiannis, David Tweedie, Elliot J. Glotfelty, Robert E. Becker, Yung‐Hsiao Chiang, Barry J. Hoffer

**Affiliations:** ^1^ Translational Gerontology Branch National Institute on Aging National Institutes of Health Baltimore MD USA; ^2^ The Ph.D. Program for Neural Regenerative Medicine College of Medical Science and Technology Taipei Medical University Taipei Taiwan; ^3^ Laboratory of Clinical Investigation Intramural Research Program National Institute on Aging National Institutes of Health Baltimore MD USA; ^4^ Department of Neuroscience Karolinska Institutet Stockholm Sweden; ^5^ Aristea Translational Medicine Corporation Park City UT USA; ^6^ Center for Neurotrauma and Neuroregeneration Taipei Medical University Taipei Taiwan; ^7^ Department of Neurosurgery Taipei Medical University Hospital Taipei Taiwan; ^8^ Department of Surgery School of Medicine College of Medicine Taipei Medical University Taipei Taiwan; ^9^ Department of Neurosurgery Case Western Reserve University School of Medicine Cleveland OH USA

**Keywords:** neurodegeneration, neuroinflammation, phenserine, preprogrammed neuronal cell death, traumatic brain injury

## Abstract

**Aim:**

Traumatic brain injury (TBI) is one of the most common causes of morbidity and mortality of both young adults and the elderly, and is a key contributing factor in about 30% of all injury‐associated deaths occurring within the United States of America. Albeit substantial impact has been made to improve our comprehension of the mechanisms that underpin the primary and secondary injury stages initiated by a TBI incident, this knowledge has yet to successfully translate into the development of an effective TBI pharmacological treatment. Developing consent suggests that a TBI can concomitantly trigger multiple TBI‐linked cascades that then progress in parallel and, if correct, the multifactorial nature of TBI would make the discovery of a single effective mechanism‐targeted drug unlikely.

**Discussion:**

We review recent data indicating that the small molecular weight drug (−)‐phenserine tartrate (PhenT), originally developed for Alzheimer's disease (AD), effectively inhibits a broad range of mechanisms pertinent to mild (m) and moderate (mod)TBI, which in combination underpin the ensuing cognitive and motor impairments. In cellular and animal models at clinically translatable doses, PhenT mitigated mTBI‐ and modTBI‐induced programmed neuronal cell death (PNCD), oxidative stress, glutamate excitotoxicity, neuroinflammation, and effectively reversed injury‐induced gene pathways leading to chronic neurodegeneration. In addition to proving efficacious in well‐characterized animal TBI models, significantly mitigating cognitive and motor impairments, the drug also has demonstrated neuroprotective actions against ischemic stroke and the organophosphorus nerve agent and chemical weapon, soman.

**Conclusion:**

In the light of its tolerability in AD clinical trials, PhenT is an agent that can be fast‐tracked for evaluation in not only civilian TBI, but also as a potentially protective agent in battlefield conditions where TBI and chemical weapon exposure are increasingly jointly occurring.

## 1 INTRODUCTION

This is a review of our work focusing on evidence for repurposing (−)‐phenserine tartrate (PhenT),^1^, ^2^ an oral drug previously safely evaluated in a phase 3 trial in Alzheimer's disease (AD), as both a treatment for, and pharmacological tool to understand, mild (m) to moderate (mod) traumatic brain injury (TBI).^3^ TBI is a leading cause of death and long‐term disability, with 10 million people annually suffering a TBI event worldwide.^4^, ^5^, ^6^ The vast majority of TBIs are mild to moderate in nature and account for 80%‐95% of cases.^7^, ^8^ In war zones, mTBI and modTBI represent a particularly difficult health issue. Military personnel, in particular, face hazards with high risks of TBI (combat injuries, blasts, operational training, and accidents). As highly active individuals, they may have prior exposures to TBI (ie, sports) before service^9^ increasing risks from re‐injury and, as veterans, they suffer a range of long‐term issues (functional impairments and disabilities) and risks as a result of earlier head injury intensifying the reaction to later head injuries suffered in aging.^10^ Of the 17,672 TBIs reported by the Department of Defense in 2016, 85.9% were classified as mild.^11^ With military personnel more likely to “push through” injury, premature return to active service is a concern,^10^ as deficits with executive functions and other unresolved sensorimotor, cognitive, and/or emotional mTBI sequelae interfere with proficiency in performing military duties.^10^ Some 85% of veterans report experiencing mTBI during their wartime service.^12^ Of equal concern, the number of civilians caught up as innocent bystanders in war zones is alarming.

With increases in survival rate following initial injury, modTBI can result in substantial and lifelong cognitive, physical, and behavioral impairments that require long‐term access to health care and disability services.^7^, ^13^ Particularly vulnerable are the elderly, in which the same insult generally causes greater disability and can result in a dramatic increase in the risk of neurodegenerative and neuropsychiatric disorders.^13^, ^14^, ^15^ Notably, military veterans have a TBI incidence 8‐ to 44‐fold that of civilians,^16^ with TBI challenges in earlier life likely driving the increased risk of Alzheimer's disease (AD)^17^, ^18^ and Parkinson's disease (PD).^19^, ^20^, ^21^

PhenT is an oral medication that is lipid soluble and readily enters the brain; it was originally developed as an anticholinesterase.^2^ It proved well tolerated in human studies (645 subjects for up to 1 year) and demonstrated a signal of efficacy in AD.^22^, ^23^, ^24^ Notably, recent studies have demonstrated a far more interesting pharmacological action, in that it inhibits programmed neuronal cell death (PNCD, eg, apoptosis/autophagy) across multiple cellular and animal neuronal injury models at clinically translatable doses.^1^, ^25^, ^26^, ^27^, ^28^ Detailed within our results described below, across preclinical neuronal injury models particularly in mTBI and modTBI, PhenT mitigates impairments in synaptic integrity, neuroinflammation and inhibits gene pathways leading to AD.^1^, ^25^, ^26^ All of these, and particularly inhibition of PNCD, are pertinent to human mTBI and modTBI.^29^, ^30^, ^31^, ^32^, ^33^

**Figure 1:**
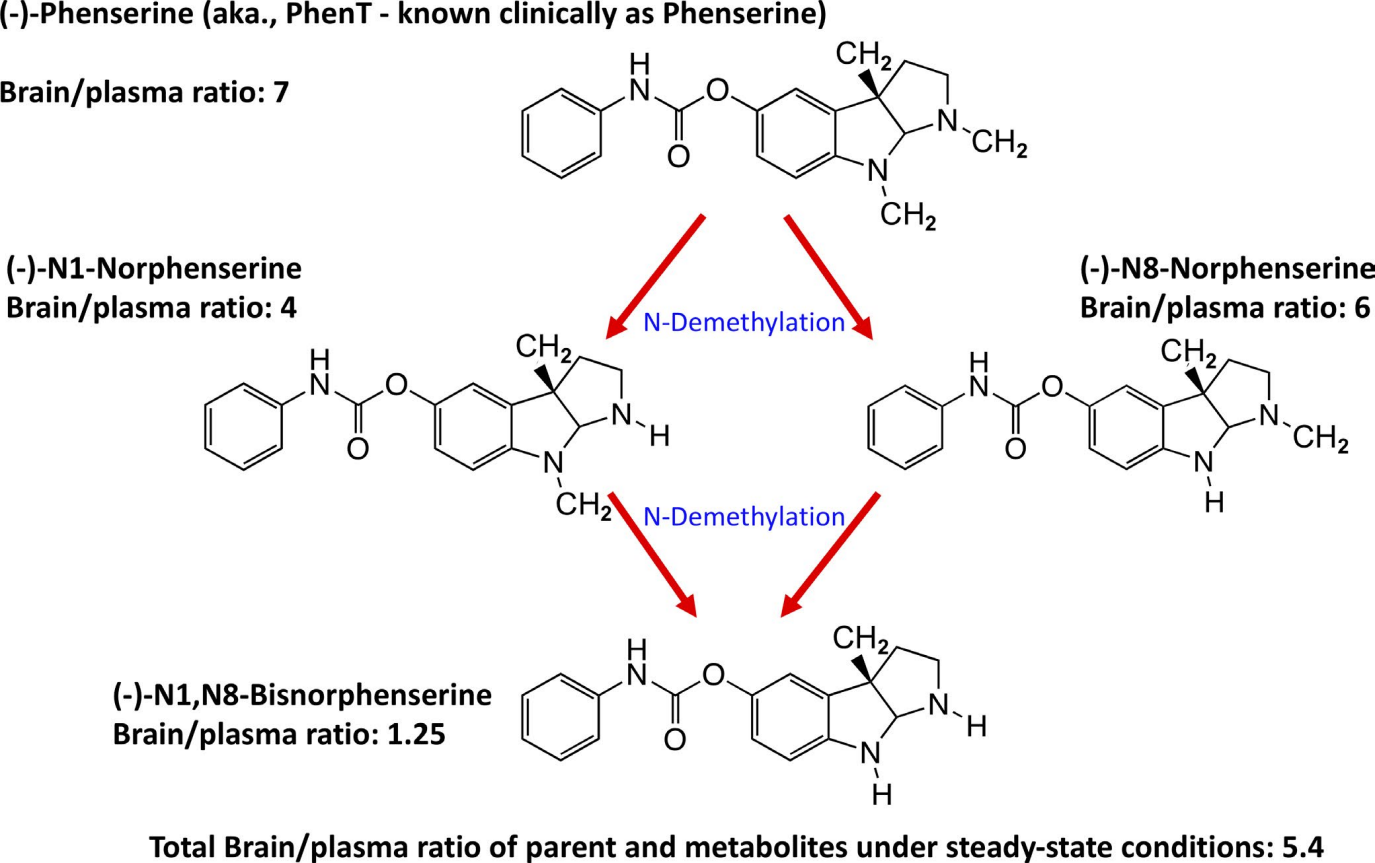
Structures and brain/plasma ratios of (−)‐phenserine (PhenT) and its three primary active metabolites. Following its administration, PhenT undergoes N‐demethylation to generate (−)‐N1‐norphenserine, (−)‐N8‐norphenserine, and, ultimately, (−)‐N1,N8‐bisnorphenserine

PhenT is a low molecular weight (mw 487.5), (−)‐ chirally pure, lipophilic (Log D 2.2) orally bio‐available agent. It and three primary active first‐pass hepatic metabolites readily enter brain. All achieve brain levels that vary between 7‐ and 1.25‐fold higher than concomitant plasma ones^1^, ^2^, ^34^ (Figure 1) and, in concentration‐dependent relationships (EC_50_s = 26 to 100 nmol/L), produce a range of pharmacological benefits relevant to TBI and AD neuropathologies. Notably, PhenT provides protection from PNCD, anti‐inflammatory, and antioxidative stress; its actions include augmenting neurotrophic factor levels, brain acetylcholine levels, and neuronal stem cell survival and differentiation, as well as inhibiting APP and Aβ generation, as recently reviewed.^1^, ^25^, ^26^, ^27^, ^28^, ^35^, ^36^, ^37^, ^38^, ^39^ PhenT is well tolerated across preclinical animal models, and in human studies (645 subjects) for up to 1 year,^3^ positively impacted markers of efficacy in AD^22^, ^23^, ^24^; importantly, it also mitigates multiple sequelae instigated by TBI and brain injury.^1^, ^25^, ^26^

### 1.1 Memory preservation in mTBI mice

**Figure 2:**
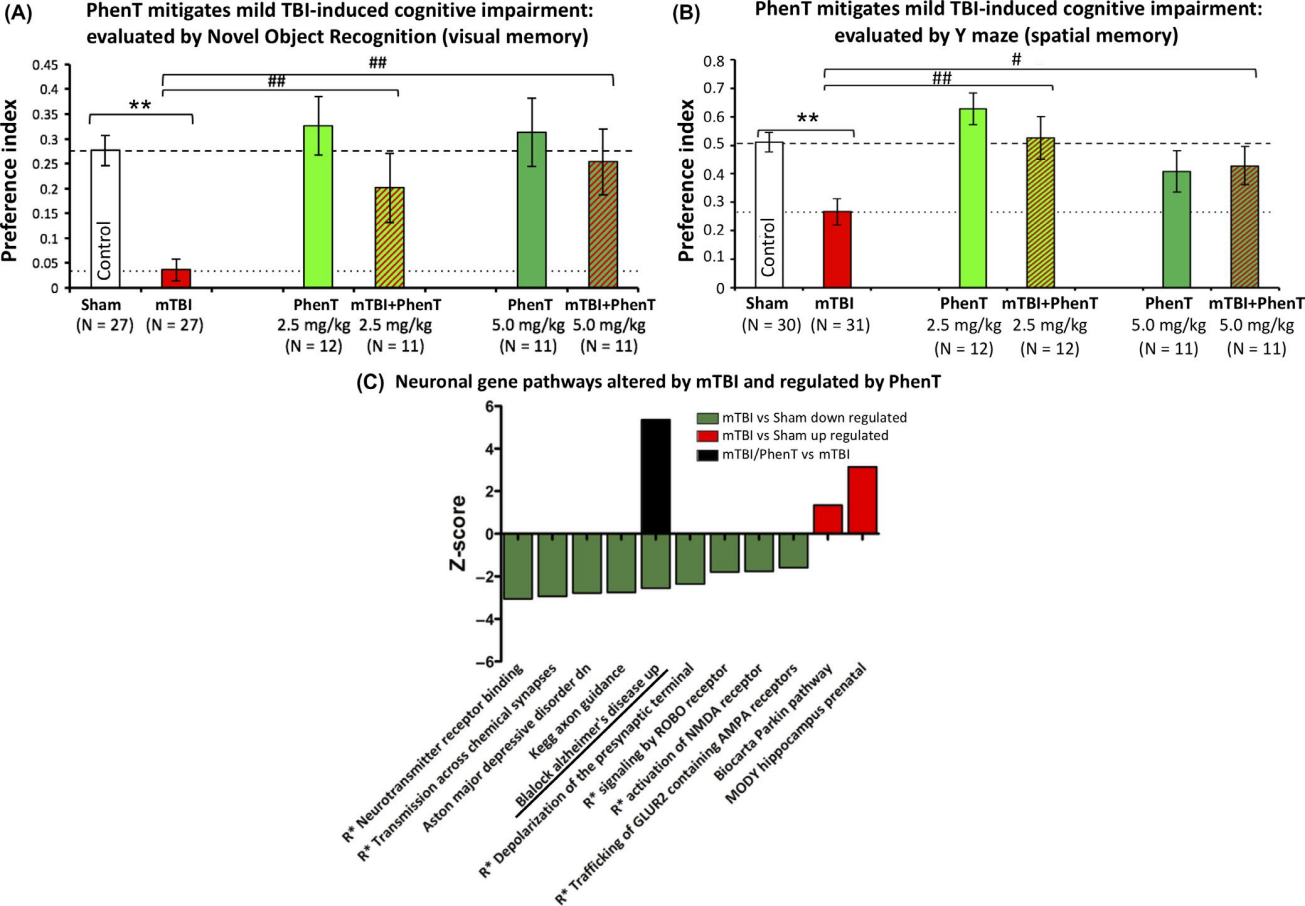
mTBI‐induces memory deficits and triggers gene pathways leading to AD that PhenT reverses. A, mTBI‐induced an impairment in visual memory (NOR) vs control uninjured (Sham) mice (**P < .01) when evaluated 7 days postinjury. PhenT significantly ameliorated this damage (both doses ##*P* < .01) when administered twice daily postinjury for 5 days and washed out for 2 days prior to cognitive evaluations. Data are mean ± SEM; one‐way ANOVA revealed a significant effect between groups (*F*(5,98) = 7.770, *P* = .000). Fisher's LSD post hoc analysis revealed that the preference index of the “mTBI” group was significantly lower than all others (##*P* < .01).^25^ B, mTBI‐induced deficits in spatial memory compared with control uninjured (Sham) animals (**P < .01), as evaluated by the Y‐maze paradigm. PhenT significantly mitigated this (^##^ *P* < .01 for 2.5 mg/kg and ^#^ *P* < .05 for 5 mg/kg). One‐way ANOVA revealed a significant effect between groups [*F*(5,105) = 6.190, *P* = .000]. Fisher's LSD post hoc analysis revealed that the preference index of the “mTBI” group was significantly lower than all other groups other than PhenT 5 mg/kg (*P < .05, **P < .01). C, CNS neuronal canonical and noncanonical pathways observed to be triggered by mTBI when compared to sham animals, and the effects of PhenT postinjury treatment (R* refers to Reactome). The majority of pathways were observed to be downregulated by mTBI, with only two that were upregulated. Two pathways were associated with neurodegenerative disease: “Blalock Alzheimer's Disease Up” and “Biocarta Parkin Pathway”. Whereas postinjury treatment of animals with PhenT had no substantial effect on the majority of pathways; notably, the AD‐related pathway was regulated by this drug. Specifically, PhenT posttreatment reversed the effects triggered by mTBI on the AD pathway^25^

In Figure 2, as evaluated by the novel object recognition (NOR) test at 7 days following a mild concussive TBI (a 30 g free falling weight from 80 cm striking a 30 g mouse on the left side of the head in the area of the parietal cerebral cortex above the hippocampus), PhenT at two clinically relevant doses (2.5 and 5.0 mg/kg BID × 5 days initiated after mTBI) mitigated mTBI‐induced cognitive impairment.^25^ mTBI impairments in spatial memory evaluated by Y‐maze also were mitigated, and increased expression of gene pathways leading to AD was reversed (Figure 2).^25^ These mTBI conditions simulate a human falling on their head from a 3‐foot fall and are considered mild and a concussive injury.^1^ Inhibiting TBI‐instigated gene pathways leading to chronic neurodegenerative disorders is important in the light of an increasing number of studies indicating that TBI associates with an increased incidence of dementia, AD, and Parkinson's disease.^13^, ^14^, ^15^, ^16^, ^17^, ^18^, ^19^, ^20^, ^21^ Important in these studies is that PhenT was dosed twice daily for 5 days initiated after injury and was washed out for 2 days prior to the evaluation of actions on TBI‐induced cognitive impairments (measured on day 7 onwards). Actions on gene pathways were evaluated in the hippocampus obtained from the injured cerebral hemisphere on day 14 postinjury; after a further 7‐day washout period. These results on cognition and the AD gene pathway can hence be interpreted as evidence for a PhenT positive effect against postinjury pathology in the absence of drug, rather than a transient symptomatic effect in the presence of drug.

Detailed below are our latest PhenT preclinical studies demonstrating inhibition of PNCD and neuroinflammation in mice challenged with mTBI; notably, this was cross‐validated in both AD transgenic (APP + PS1) mice subjected to mTBI^40^ and in controlled cortical impact (CCI) injury challenged mice,^41^ which is a well‐characterized animal model of modTBI.

### 1.2 PhenT mitigates mTBI‐induced neurodegeneration

**Figure 3:**
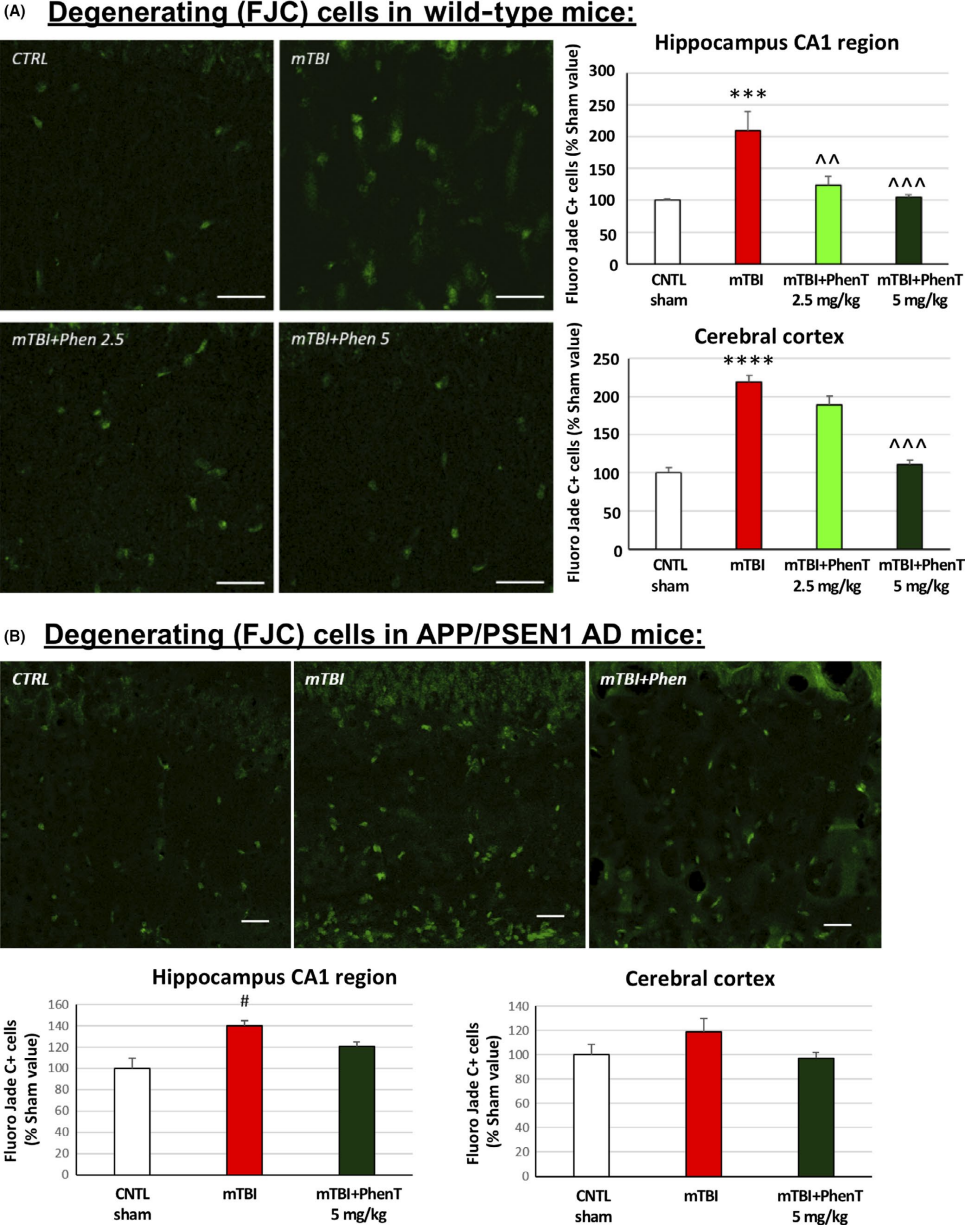
PhenT prevents mTBI‐induced neurodegeneration (72 h post‐mTBI). After mTBI injury, evaluation of FJC + cells (green appearing cells) showed a significant increase in the No. of degenerating neurons in mTBI vehicle mice across all studied brain areas vs control (CTRL), sham mice for both (A) young wild type (WT) and (B) APP + PS1 AD transgenic mice. PhenT (5 mg/ kg, BID) fully counteracted mTBI‐induced neurodegeneration. Representative images of FJC staining in hippocampus CA1. Data are mean ± SEM, magnification ×60, scale bar = 30 μm. For (A) WT mice: n = 5/group; *P < .05, **P < .01, ***P < .001 vs CTRL by Tukey's post hoc test; ^^*P* < .01 vs mTBI alone by Tukey's post hoc test. For (B) APP + PS1 AD mice: n = 5/group, #*P* < .05 vs CTRL (sham) group Mann‐Whitney rank test

To evaluate the basis of the PhenT mediated mitigation of mTBI‐induced cognitive impairments, the antiapoptotic potential of PhenT was appraised in the brains of separate cohorts of mice. Using immunohistochemical techniques detailed previously,^40^, ^41^, ^42^, ^43^ PNCD was evaluated at 72 hours following head injury by Fluoro‐Jade C (FJC) staining and counting of degenerating cells within the parietal cortex and the CA1, CA3, and dentate gyrus regions of the hippocampus in sham (uninjured)‐, mTBI (vehicle treated)‐, and mTBI + PhenT (2.5 and 5 mg/kg, BID)‐treated animals. As illustrated in Figure 3A, the mTBI‐induced PNCD was dose‐dependently mitigated by PhenT across all evaluated brain areas.^40^ To cross‐validate this in a more complex model in which neuronal cell dysfunction and death, together with neuroinflammation, were already occurring, aged (10‐12 months old) APP + PS1 AD transgenic (Tg) male mice were challenged with mTBI (a similar concussive 30 g weight drop), followed by identical PhenT or vehicle posttreatment. As shown in Figure 3B, the background level of cell death evident from FJC staining was greater than in WT mice across all brain regions, in those with and without mTBI. mTBI demonstrated a similar trend to elevate FJC‐positive (+) staining cells, and PhenT mitigated this.

### 1.3 PhenT reduces neuroinflammation in mTBI mice

**Figure 4:**
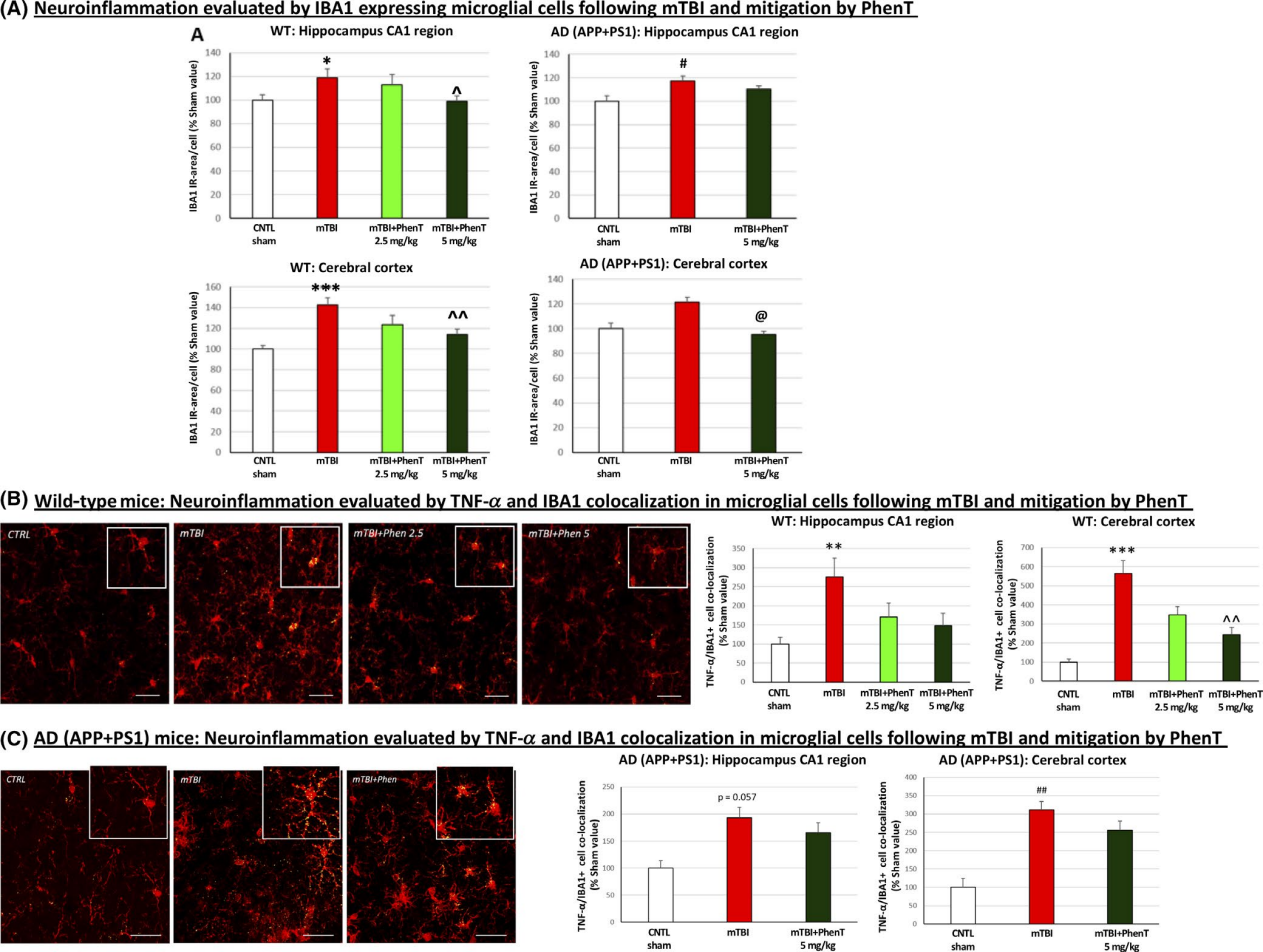
PhenT mitigates markers of microglial neuroinflammation in mTBI‐challenged WT and AD mice across hippocampus and cerebral cortex (72 h post‐mTBI). After mTBI injury, evaluation of (A) IBA1 + cells (red in appearance) showed a rise in number in mTBI vehicle treated‐WT and AD vs control (CTRL) mice (w/o mTBI). PhenT mitigated this. A mTBI‐induced elevation in microglial cells co‐expressing TNF‐α (yellow in appearance) was also evident in (B) WT and (C) AD Tg mice, which was mitigated by PhenT treatment (n = 5/group). In the higher magnification insets in (B) and (C) IBA1 and TNF‐α are red and yellow, respectively (the TNF‐α is evident in small yellow punctate within the red colored IBA1 immunostained microglia). *P < .05, **P < .01, ****P < .0001 vs CTRL by Tukey's post hoc test; ^*P *< .05, ^^*P* < .01, ^^^*P *< .001, vs mTBI by Tukey's post hoc test. #*P *< .05 ##*P *< .01 vs CTRL by Mann‐Whitney rank test; @*P *< .05 vs mTBI by Mann‐Whitney rank test*.* Data shown as mean ± SEM Scale bar = 30 μm^40^

Neuroinflammation was evaluated by counting activated microglial cells expressing the marker IBA1 as well as by counting microglial cells co‐expressing IBA1 and immunoreactivity for the pro‐inflammatory cytokine TNF‐α (using anti‐IBA1 antibody: Abcam; anti‐TNF‐α antibody: Abbiotec). As evident in Figure 4A, activated microglial (IBA1 positive (+) staining) cells—a classical marker of neuroinflammation—were elevated in WT mice challenged with mTBI. PhenT posttreatment mitigated this mTBI‐induced effect dose‐dependently. Likewise, mTBI challenge induced a rise in IBA1 + activated microglial cells expressing the pro‐inflammatory marker TNF‐α (Figure 4B), which was similarly mitigated by PhenT posttreatment. This was likewise cross‐validated in the more complex model of aged (10‐12 months old) APP/PS1 AD Tg male mice (Figure 4C) (within Figure 4B,C IBA1 is red and TNF‐α yellow punctates, as evident in the higher magnification insets). Notably, the background number of activated (IBA1+) microglia was greater than in WT mice across brain regions, in those with and without mTBI. mTBI further elevated this, and a similar treatment effect was achieved with PhenT (5 mg/kg BID).^40^ Not shown, but clearly apparent, PhenT also significantly mitigated TBI‐induced astrocytosis as evaluated glial fibrillary protein (GFP) immunohistochemistry (see data and figures within Ref.^40^).

### 1.4 PhenT mitigates mTBI‐induced losses in synaptic integrity

**Figure 5:**
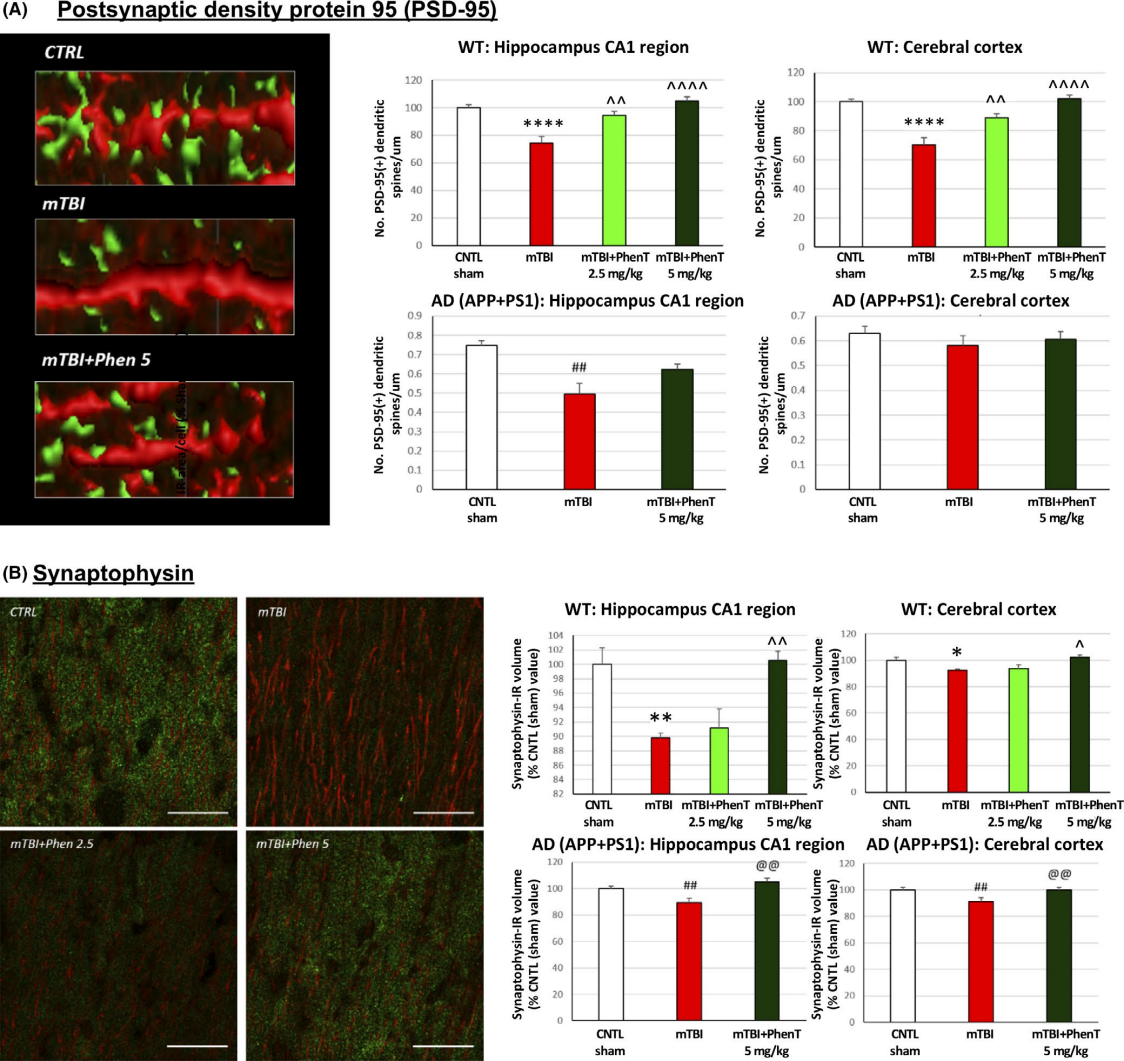
PhenT mitigates mTBI‐induced reductions in pre‐ and postsynaptic markers of synaptic integrity (72 h post‐mTBI) in both WT and AD mice: A, PSD‐95: mTBI induced a loss of PSD‐95 + dendritic spines across all analyzed areas in WT mice, and in hippocampus of AD mice, vs the sham (CTRL) group. In contrast, PhenT‐treated mTBI mice were not statistically different from the sham (CTRL) group. Specifically, WT mTBI mice treated with PhenT (2.5 and 5 mg/kg) possessed a greater number of PSD‐95 + dendritic spines across both hippocampus and cortex, vs the mTBI vehicle group. Representative images of PSD‐95 + spines (green) in MAP2 + dendrites. Data are expressed as No. of PSD‐95 + dendritic spines/μm. B, Presynaptic synaptophysin: the total volume occupied by synaptophysin immunoreactivity (IR) was quantified across WT and AD (APP/PSEN1) mice and found to be significantly decreased in the mTBI vehicle group, vs the respective sham (CTRL) group. By contrast, mTBI PhenT‐treated animals had levels no different from sham (CTRL) mice. Notably, PhenT treatment resulted in significantly higher levels of synaptophysin IR, vs the mTBI vehicle group, across all analyzed brain areas in both WT and AD mice. n = 5 per group. *P < .05, **P < .01, ****P < .001 vs CTRL by Tukey's post hoc test; ^*P *< .05, ^^*P *< .01, ^^^^*P *< .0001 vs mTBI by Tukey's post hoc test. ##*P *< .01 vs CTRL by Mann‐Whitney rank test. Data are mean ± SEM values. @@*P *< .01 vs mTBI by Mann‐Whitney rank test. Scale bar = 20 μm

To evaluate whether mTBI‐induced cognitive deficits and elevations in markers of cell loss (FJC) and neuroinflammation (TNF‐α and IBA1) are allied with a loss of synaptic integrity (which associates with cognitive loss^44^), post‐ and presynaptic protein markers of synaptic integrity (the postsynaptic density‐95 (PSD‐95) and the presynaptic marker synaptophysin, respectively) were immunohistochemically quantified in control (w/o mTBI) and mTBI‐challenged mice posttreated with vehicle or PhenT. Shown in Figure 5, mTBI reduced post‐ and presynaptic markers of synaptic integrity across all studied brain areas in WT as well as AD (APP + PS1) Tg mice. Notably, PhenT posttreatment inhibited this. The biological relevance of mitigating mTBI‐induced losses in pre‐ and postsynaptic proteins was evaluated by electrophysiology by quantifying long‐term potentiation (LTP) in hippocampal brain slices acquired from a separate cohort of sham and mTBI mice treated with either vehicle or PhenT (5 mg/kg, BID) and measured at 72 hours (the same time that synaptic protein changes were determined). An impairment in the expression of LTP was evident in mTBI vehicle‐administered mice, coinciding with mTBI‐induced declines in synaptic proteins and, notably, this was mitigated in mTBI‐challenged animals administered PhenT postinjury.^40^

Long‐term potentiation (LTP) results in the long‐lasting enhancement of synaptic strength consequent to repetitive activation of central glutamatergic synapses and is critical to memory encoding.^45^, ^46^ LTP impairment is associated with memory deficits in rodents,^47^ has previously been noted to occur following TBI,^48^, ^49^ and hence its mitigation by PhenT is potentially valuable as LTP is regarded as a necessity for hippocampal processing.

### 1.5 Extracellular vesicles as brain biochemical probes

It is now widely accepted that TBI represents a time‐dependent process, rather than a single event, and can involve the progression of multiple cascades that can amplify one another and detrimentally impact the surrounding neural microenvironment. This process may in some individuals lead to early dementia onset,^50^ but predicting which individuals are the most vulnerable and the sequence of TBI‐induced cellular cascades that underpin this has been difficult to determine in the absence of easy to sample, well‐validated biomarkers. Analysis of neuronal‐ and astrocyte‐derived extracellular vesicles (often termed exosomes) sampled from plasma can provide direct information about neurodegenerative and neuroinflammatory pathophysiology.^51^ Extracellular vesicles, 50‐200 nm‐sized membranous vesicles, are generated and released by most viable cells and accumulate in biological fluids, including the blood, where they can be sampled in a time‐dependent manner. The careful selection of the cell type from which extracellular vesicles derive, by use of defined markers expressed on their surface—such as L1CAM (an adhesion molecule predominantly expressed on neuron‐derived extracellular vesicles) or of the protein glutamine aspartate transporter (GLAST) (expressed on astrocyte‐derived extracellular vesicles) allows the immunoprecipitation of extracellular vesicles that are enriched for neural or astrocytic origin, respectively.^50^, ^51^ Analysis of the contents of such extracellular vesicles provides a window to the contents of neural cells within the brain in relation to their response to physiological challenges, disease, and its progression and responses to drugs.^51^, ^52^ In this regard, extracellular vesicles are proving increasingly valuable as a source of potential biomarkers to predict TBI outcomes.^53^, ^54^, ^55^ However, as they are key role players in implementing intercellular communication and initiating physiological responses,^56^, ^57^ the contents of extracellular vesicles can additionally be valuably applied to drug screening.

**Figure 6:**
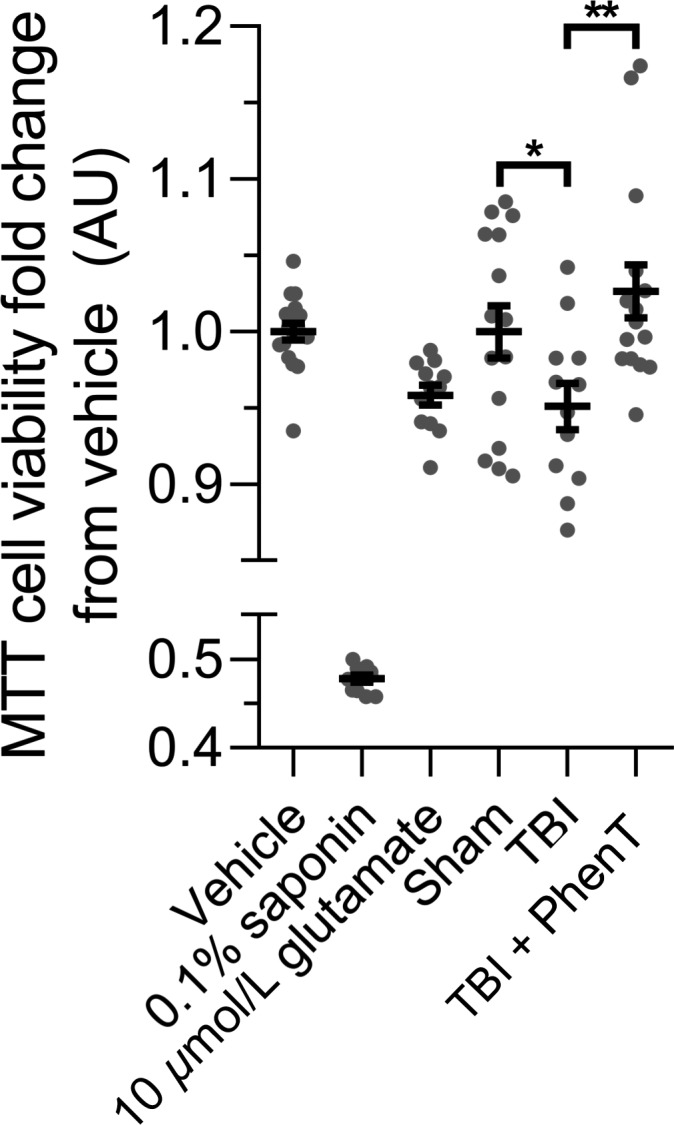
PhenT inhibits the neuronal viability decrease induced by TBI astrocyte‐derived extracellular vesicles. E18 rat cortical neurons were seeded in a 96‐well plate at 100 000 cells/well. At 14 DIV, cells were treated with astrocyte‐derived extracellular vesicles (AEV) immunoprecipitated using antibodies targeting the astrocyte cell surface antigen‐1 (anti‐GLAST) from the plasma of mice subjected to sham or mTBI (from a 30 g weight drop) with or without PhenT (5 mg/kg, BID) posttreatment. Treatments were carried out in triplicate at a concentration of 600 EVs/neuron for 48 h. After treatment, neurons were subjected to the MTT cell viability assay. Neurons treated with 0.1% saponin detergent and 10 μmol/L glutamate were used as positive controls of neurotoxicity (and induced substantial cellular loss). No significant differences in total EV or AEV number was noted in plasma samples obtained across the sham, mTBI and mTBI PhenT groups. Number of mice per group: sham, n = 5; TBI, n = 4; TBI + PhenT, n = 5. Statistical analysis: two‐tailed unpaired t‐tests with a confidence interval (CI) of 95%; *P = .0469, **P = .0039. Specifically, AEVs obtained from mTBI‐challenged mice administered vehicle (saline) resulted in a statistically significant loss (*P *= .0469) in the viability of cultured cortical neurons, as compared to sham mice that were not subjected to mTBI (evaluated by MTT assay). In contrast, AEVs derived from mTBI mice administered PhenT postinjury resulted in a cortical neuron viability that was no different from sham (*P*> .05), but, notably, was significantly greater than mTBI vehicle (*P* = .0039)

As astrocytes provide trophic support of neurons that can be altered in the disease state, we evaluated whether astrocyte‐enriched (GLAST) extracellular vesicles derived from a pathological state, specifically mTBI in mice, could adversely impact neuronal survival of primary neuronal cultures. Specifically, embryonic (18 days) rat cortical neurons in culture were incubated with plasma‐derived astrocyte‐enriched extracellular vesicles from control sham mice and compared to those obtained from mice subjected with mTBI (30 g weight drop) 24 hours earlier. As illustrated in Figure 6, those incubated with extracellular vesicles from mTBI mice had a significantly (*P *< .05) reduced survival. Notably, reduction in survival was completely reversed by the postadministration of PhenT (5 mg/kg BID) to mice, in accord with the described actions of PhenT mitigating PNCD, astrocytosis, and neuroinflammation. This demonstrates a functional role associated with pathologically induced changes in the cargo of astrocyte‐enriched extracellular vesicles obtained from a plasma sample, and drug‐mediated mitigation.

### 1.6 PhenT mitigates PNCD, neuroinflammation and behavioral deficits in CCI modTBI

**Figure 7:**
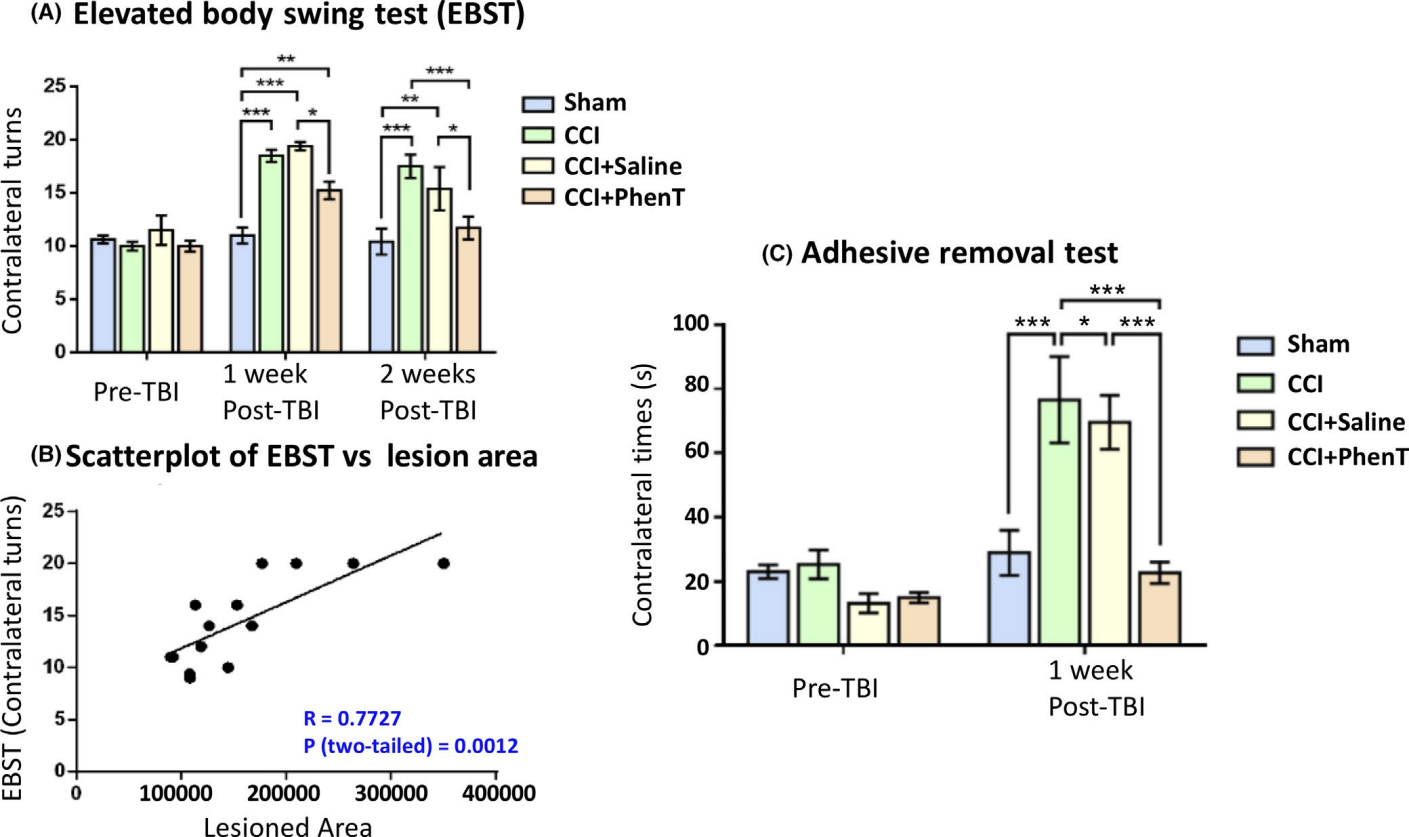
PhenT mitigates modTBI‐induced impairments in Elevated Body Swing Test (EBST) (impairments in which associate with greater TBI‐induced lesion size) as well as in Adhesive removal test (a measure of sensory/motor neglect). PhenT treatment (2.5 mg/kg, BID × 5 days post‐CCI) improved motor asymmetry, as revealed by behavioral measurement and correlation between behavioral evaluation and lesion size.^41^ A, Motor asymmetry measured by EBST. Analysis by two‐way ANOVA followed by Bonferroni *t*‐test. Data are mean ± SEM; n = 4/group (SHAM, CCI, CCI + Saline), n = 7 (CCI + PhenT). *P < .05, **P < .01, ***P < .001, compared among each group. B, Correlation between EBST and contusion lesion area was significant within Sham, CCI, CCI + Saline, CCI + PhenT groups at 2 wk after CCI. Analysis by Pearson's Correlation. n = 14, *r* = .7727, *P* (two‐tailed) = .0012. (C) Adhesive removal test: PhenT ameliorated sensory/motor neglect induced by CCI.^41^ CCI produces altered sensor/motor function, shown by spending more time to remove a sticker from the contralateral forepaw evaluated 1 wk after TBI in comparison to pre‐TBI (PRE). PhenT treatment (2.5 mg/kg BID × 5 days post‐CCI) significantly reduce this deficit. Analysis by two‐way ANOVA followed by Bonferroni *t*‐test. Data are mean ± SEM; n = 4 (Sham, CCI, CCI + Saline), n = 7 (CCI + PhenT). *P < .05, ***P < .001, compared among each group

**Figure 8:**
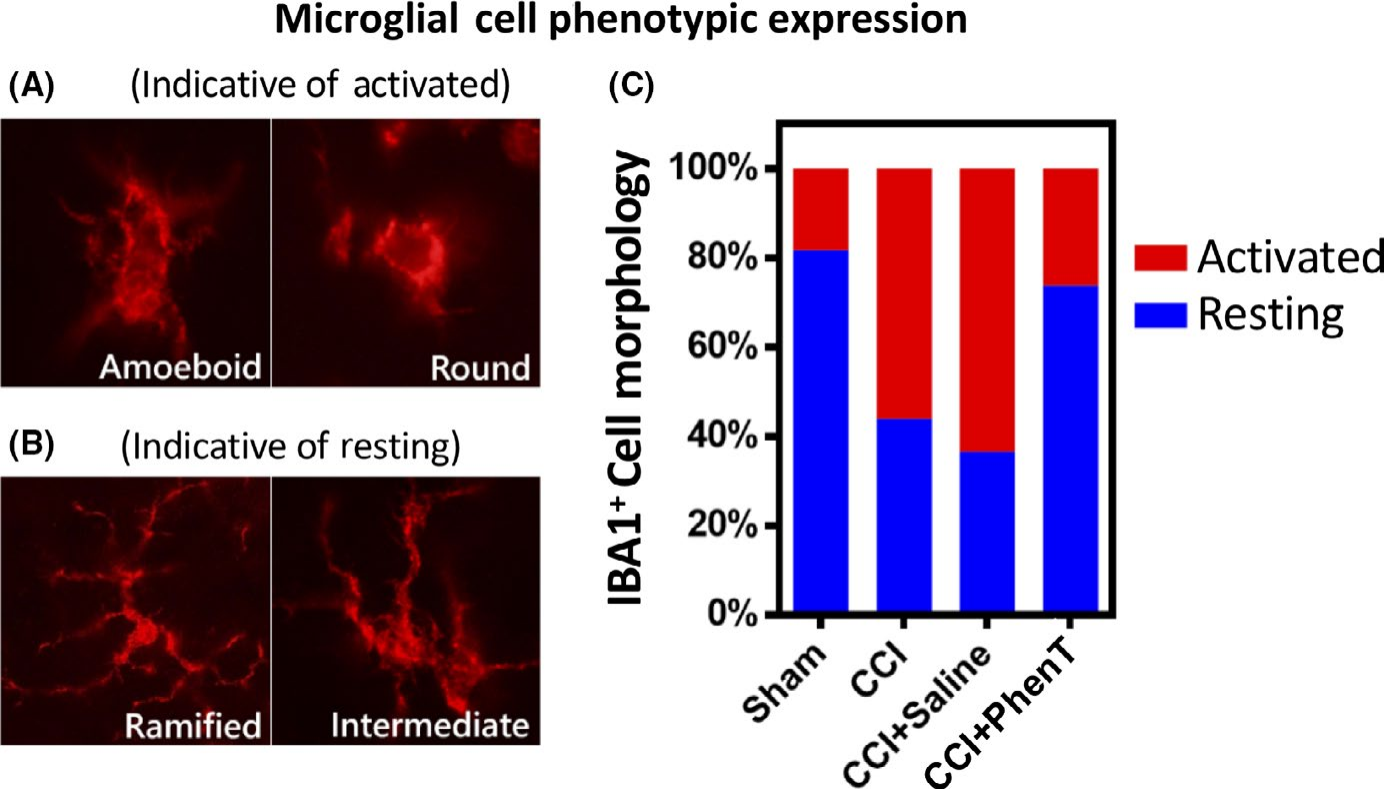
PhenT mitigates neuroinflammation induced by moderate CCI TBI, as assessed by IBA1 cell morphology ratio of resting vs activated microglial cells in brain tissue adjacent to the contusion area. Moderate TBI induced the activation of microglial cells, as evaluated in tissue adjacent to the contusion area by IBA1 immunostaining, and PhenT (2.5 mg/kg BID × 5 days after CCI) fully mitigated this. Representative IBA1 immunofluorescence stained microglial cells that are “activated” (ie, reactive) with ameboid and round forms (A) (microglial activation results in a retraction of their processes, which become fewer and much thicker, an increase in the size of their cell bodies, a change in the expression of various enzymes and receptors, and production of immune response molecules; for example, TNF‐α, etc. Some microglial cells return into a proliferative mode, and microglial numbers around the lesion site start to multiply). Also shown are “resting” IBA1 stained microglia with a ramified and intermediate (B) morphology (characterized by a small cell body and much elaborated thin processes, which send multiple branches and extend in all directions). (C) Quantification of the proportions of microglia into activated (reactive) and resting stages. Notably, PhenT significantly reduced the active (reactive) form of the microglia (*P* = .022) compared with CCI + saline group (from 64.3 ± 3.39% (CCI + saline) to 25.1 ± 3.59% (CCI + PhenT)). This data cross‐validates the anti‐inflammatory actions that PhenT demonstrates in mild TBI (Figure 4)

**Figure 9:**
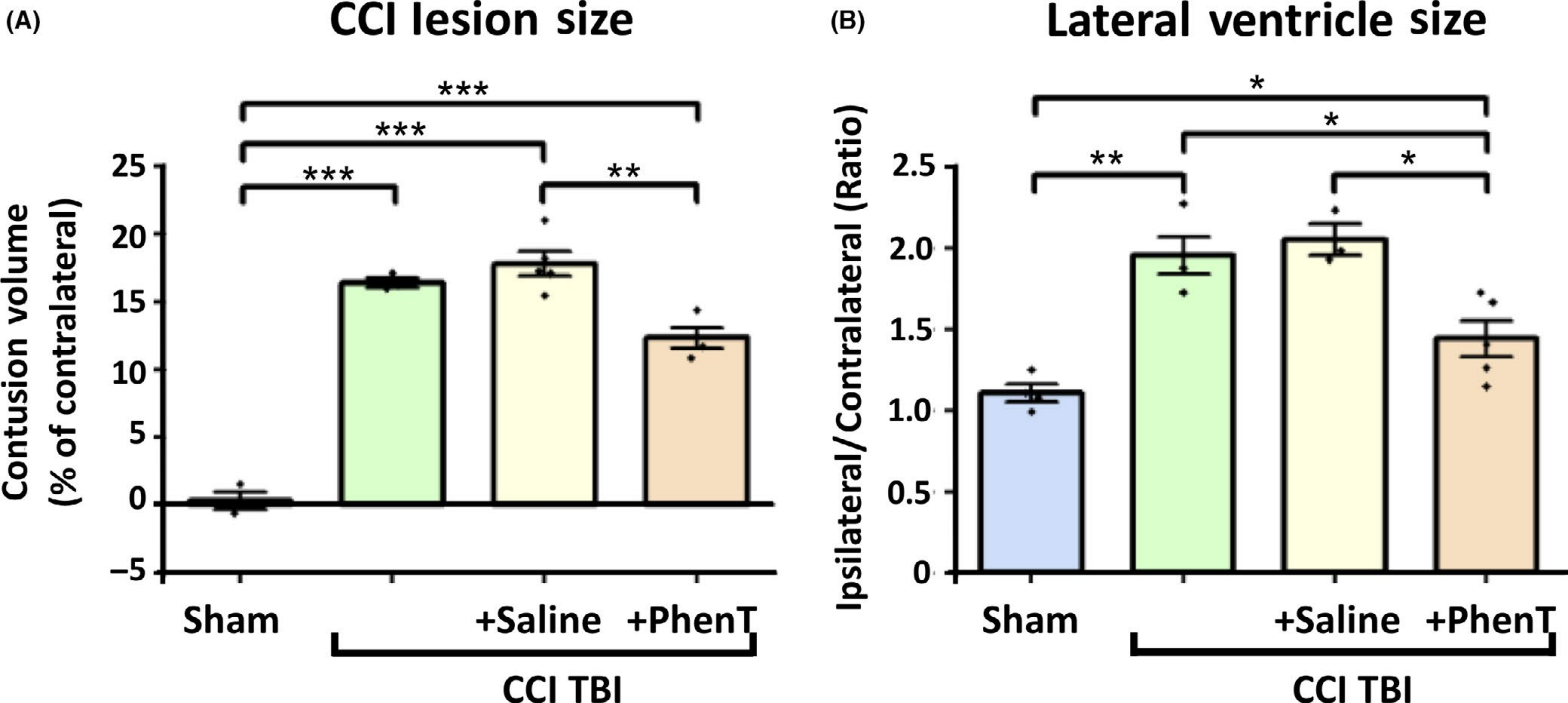
Lesion size and lateral ventricle size. PhenT treatment (2.5 mg/kg, BID × 5 days after CCI) reduced lesion volume (A) and lateral ventricle (LV) swelling (B) evaluated 2 wk after CCI. Lesion volume was quantified using IMAGE‐PRO PLUS 6 software (Media Cybernetics).^41^ (A) Significant reduction of lesion size was found in the PhenT group. *P < .05, ***P < .001, vs vehicle‐treated and CCI‐only groups. (B) Significant differences in LV size ratio between ipsilateral and contralateral sides were also found between PhenT‐ and saline‐treated groups (*P < .05). Analysis by one‐way repeated measure ANOVA followed by Holm‐Sidak method. Data are mean ± SEM; n = 4 (SHAM, CCI, CCI + Saline), n = 7 (CCI + PhenT)

No single animal model mimics all aspects of human TBI owing to the heterogeneity of clinical TBI. To successfully develop compounds for clinical TBI, evaluation in several TBI models and injury severities is warranted at clinically translatable doses for predictive value.^58^ CCI is a commonly used and well‐characterized model of experimental TBI that employs a commercially available pneumatic CCI device to generate injury.^59^, ^60^, ^61^, ^62^, ^63^, ^64^ CCI injury can be scaled to produce graded TBI ranging from mild to severe. ModTBI was induced by us in WT mice to create a primary lesion in brain. This initiates the previously described secondary process of neuronal cell dysfunction and death (involving PNCD, neuroinflammation, glutamate excitotoxicity, oxidative stress) that are targets for drug therapies.^41^, ^49^, ^63^, ^65^, ^66^, ^67^, ^68^, ^69^ PhenT, evaluated at a clinically translatable dose of 2.5 mg/kg BID, substantially mitigated CCI modTBI‐induced behavioral impairments (elevated body swing test (EBST)^70^ and adhesive removal test^71^: Figure 7).^41^ Moreover, PhenT significantly and substantially mitigated neuroinflammation. As illustrated in Figure 8, CCI‐induced modTBI resulted in a phenotypic change in microglial cell morphology and number vs sham (unchallenged animals), resulting in an activated condition that is sometimes termed a M1 reactive state. PhenT at a clinically translatable dose reversed this, in line with anti‐inflammatory actions demonstrated in other models,^38^, ^40^ and reduced the percent of microglia expressing an activated state from 64.3 ± 3.39% in CCI + saline animals to 25.1 ± 3.59% in CCI + PhenT mice.^41^In accord with this, PhenT significantly reduced astrogliosis and neuronal loss in the same animals,^41^ which was reflected in a reduction in the CCI TBI‐induced contusion volume and size of the lateral ventricles (Figure 9).^41^

**Figure 10:**
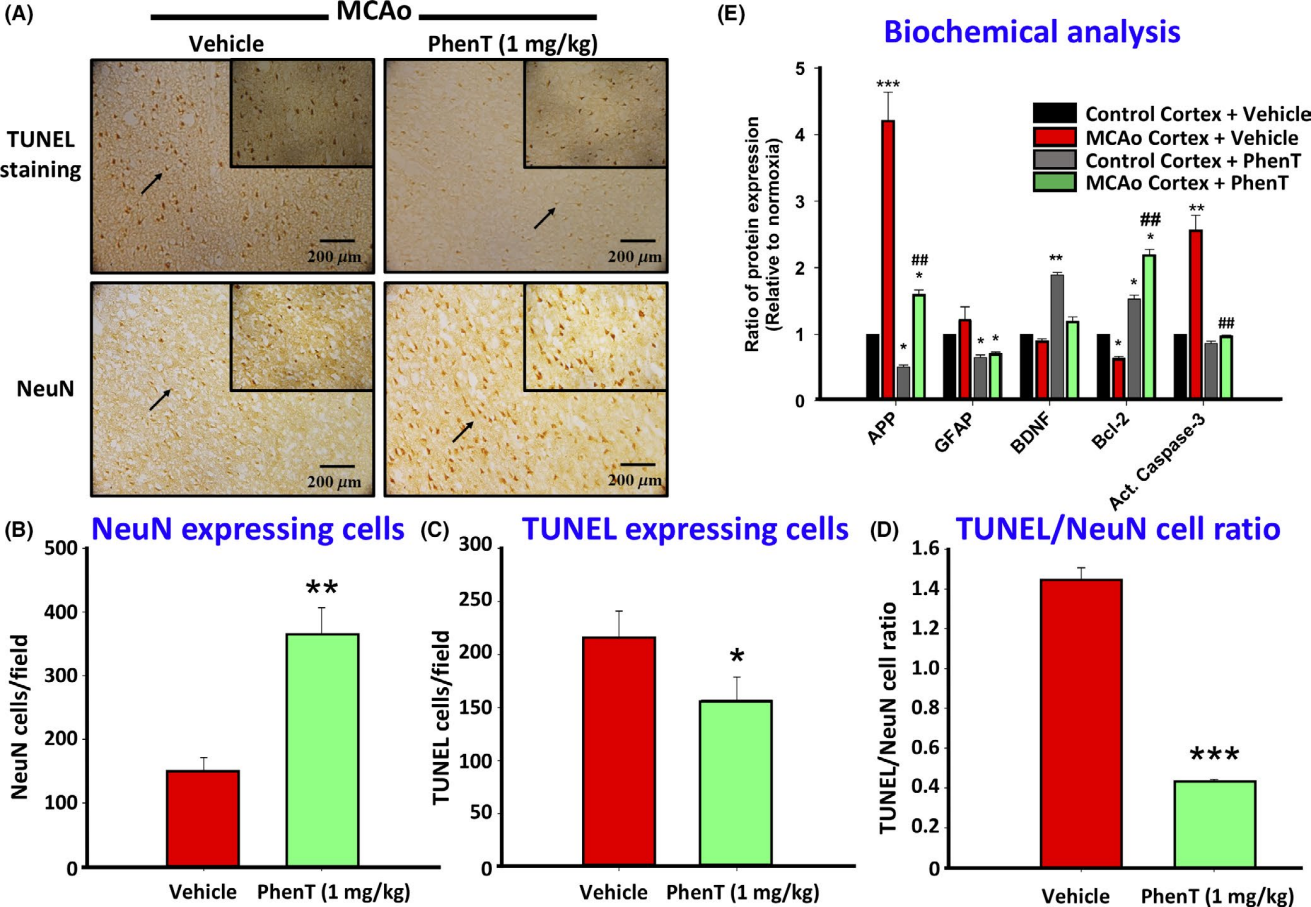
PhenT (1 mg/kg, 24 h prior to MCAo) decreased neuronal cell apoptosis/PNCD in the cortex induced by transient (60 min) MCAo in rat: If a candidate drug mitigates PNCD in one animal model of brain injury (eg, PhenT in mTBI and modTBI), in theory it should do the same across animal models of brain injury. To test this notion, PhenT was evaluated in a classical model of cerebral anoxia (MCAo in rat).^26^ (A) Representative immunohistochemistry demonstrating TUNEL staining and NeuN. Quantitative evaluation of (B) NeuN (neuronal marker) + cells, (C) TUNEL + cells, (D) TUNEL/NeuN cell ratio across similar size fields in penumbra of MCAo animals, (E) biochemical analysis of key PNCD proteins (N = 3/ group, means ± SEM, * comparison to control vehicle: *P ≤ .05, **P ≤ .01 and ***P ≤ .001; ^#^ comparison MCAo vehicle to MCAO PhenT: ^##^ *P *≤ .01).^26^ Across measures, PhenT mitigated ischemia‐induced neuronal cell loss, TUNEL staining and changes in PNCD‐related proteins

PhenT mitigation of TBI‐induced cellular loss is consistent with inhibition of PNCD, as evaluated in cellular studies and models of hypoxia.^26^ This is pertinent as cerebral ischemia commonly occurs early after TBI, may persist for up to a week after mild to moderate injury, and can further induce neuronal dysfunction and death.^72^, ^73^, ^74^ As an example, Figure 10 illustrates the efficacy of PhenT to block neuronal PNCD in the brain penumbra of the ischemic area generated by transient middle cerebral artery occlusion (MCAo) in rat.^26^ This was evaluated by a number of techniques, including TUNEL staining which is widely used to identify and quantify apoptotic cells. Our biochemical analyses, in the same study, notably additionally demonstrated that PhenT significantly elevates levels of antiapoptotic proteins (Bcl‐2) and neurotrophic factors (BDNF) and lowers proapoptotic proteins (activated caspase‐3) as well as amyloid precursor protein levels,^26^ thereby cross‐validating such actions in our previous studies.^28^, ^35^, ^37^

Thus, our studies across cellular and animal models,^1^, ^25^, ^26^ together with the data above,^40^, ^41^ indicate that PhenT inhibits PNCD (evaluated histochemically and biochemically), improves cognitive/motor outcomes, and can hence act as a probe in human TBI studies. We expect that PhenT reductions in brain injury‐induced PNCD, in large part, correlate with the lowered neuroinflammation and loss of synaptic integrity that we demonstrate. These actions, separately and together, are of potential interventional value in clinical TBI.

## 2 CONCLUSION

In synopsis, we propose that weight drop‐induced mTBI, CCI‐induced modTBI, together with anoxia (transient MCAo) models, and mechanistic study data from cultured human immortal neuronal cells and rodent primary cortical cultures prove consistent with PhenT preventing neuronal self‐induced programmed cell death. This was appraised by multiple complimentary techniques, including quantitative TUNEL, NeuN, and FJC staining, as well as Western blot quantification of pro‐apoptotic (activated caspase 3) and antiapoptotic (Bcl‐2 and BDNF) protein levels. In addition to these reviewed studies, PhenT has presented PNCD inhibiting actions in rodents challenged with toxic doses of soman, reducing its lethality and, importantly, protecting the brain from neuronal loss.^27^ In evaluating mechanisms in the soman study, PhenT was found to regulate a core set of neuroprotective genes termed 'activity‐regulated inhibitor of death' (AID) genes, as well as genes impacting neuroinflammatory pathways. The expressions of the AID genes appear to be contingent on nuclear calcium signaling, are probably CREB target genes, and seem to foster neuronal survival in both cellular and animal studies*.* Interestingly, PhenT is one of very few drugs that appears to provide protection against both TBI and organophosphorus nerve gas agents,^25^, ^27^, ^40^, ^41^ challenging insults that, in some regions of the world, co‐occur.

Together, these studies demonstrate that clinically relevant doses of PhenT impart a unique and broad range of beneficial pharmacological actions that positively impact the PNCD and neuroinflammation that results following a TBI, with the added potential of reversing key pathways associated with chronic neurodegenerative disorders such as AD. It is now appreciated that multiple processes combine together following a brain insult (whether an acute TBI or a chronic degenerative disorder like AD or PD) to initiate a self‐sustaining cycle of events leading to synaptic and neuronal dysfunction and, ultimately, cellular demise. The PhenT mitigation of (a) oxidative stress, glutamate excitotoxicity, (b) inflammation either directly or via cholinergic mechanisms, (c) APP/Aβ over‐expression, as well as ability to augment neurosphere and neuronal survival, endogenous trophic factors like BDNF and stimulate other such mechanisms,^1^, ^25^, ^26^, ^27^, ^28^, ^35^, ^36^, ^37^, ^38^, ^39^, ^40^, ^41^ provides a means to both limit cell death and maximize endogenous regenerative mechanisms. The biochemical cascades that underlie such pharmacological actions appear to be triggered by PhenT and its primary metabolites, and mediated via pathways that include the PKC and MAPK/MEK1/2 cascades,^28^ as selective inhibition of these results in a loss of neuroprotection. PhenT additionally appears to regulate key proteins (APP and α‐synuclein) posttranscriptionally,^35^, ^37^, ^75^ as well as core gene sets (eg, AID and Blalock Alzheimer's Disease Up) involved in neuronal survival and neurodegeneration.

TBI clinical trials of experimental drugs that act via a single mechanism, such as anti‐inflammatory or glutamate lowering approaches, have failed to tackle the full scope of injury‐triggered cascades that ultimately lead to neuronal loss and cognitive impairment. PhenT’s activation of multiple pathways provides the potential to support neuroprotective and neuro‐reparative mechanisms of relevance to multiple coinciding pathological processes instigated by TBI, which are likely different across human individuals that suffer a TBI event. Furthermore, it may be possible to follow key markers of these pathways in plasma sampled extracellular vesicles enriched for neuronal and astrocytic origin,^50^, ^51^, ^52^, ^53^, ^54^ and evaluate whether they are altered by PhenT treatment. In the light of its prior demonstration of safety in long‐term human studies, PhenT warrants evaluation in human TBI.

## CONFLICTS OF INTEREST

REB is an inventor on a patent related to the use of PhenT for the treatment of neurological disorders. All other authors declare no conflicts of interest.
